# Screening performance of quantitative blood loss for postpartum anemia among patients without baseline anemia

**DOI:** 10.1016/j.xagr.2026.100688

**Published:** 2026-08-29

**Authors:** Sebastian Reyes Lizaola, Ivy Huynh, Lucia Di Francesco, Swati Kumari, Tajudeen Dabiri

**Affiliations:** 1 Department of Obstetrics and Gynecology, BronxCare Health System, Bronx, NY (Reyes Lizaola, Di Francesco, Kumari, Dabiri); 2 American University of the Caribbean School of Medicine, Cupecoy, Sint Maarten (Huynh)

**Keywords:** blood loss measurement, hemoglobin decline, postpartum care, postpartum hemoglobin, postpartum laboratory testing

## Abstract

**BACKGROUND:**

Quantitative blood loss (QBL) is widely used to identify postpartum hemorrhage. Although higher blood loss is expected to be associated with greater hemoglobin (Hb) decline, the strength of this relationship and the diagnostic performance of commonly used thresholds for postpartum anemia are not well defined. Further evaluation is needed to define the utility and limitations of QBL in the postpartum assessment.

**OBJECTIVE:**

To evaluate the association between QBL and postpartum hematologic outcomes, including postpartum anemia and Hb decline, and to assess the diagnostic performance of commonly used QBL thresholds.

**STUDY DESIGN:**

This retrospective cohort study included singleton deliveries at an urban safety-net hospital in 2023 with documented QBL and available baseline and postpartum Hb values. Patients with baseline anemia, transfusion before postpartum Hb measurement, or conditions expected to substantially alter bleeding risk or Hb levels independently of delivery blood loss were excluded. Multivariable logistic regression was used to evaluate associations between QBL and postpartum anemia, defined as Hb<10 g/dL, and multivariable linear regression was used to evaluate Hb decline. Diagnostic performance of QBL thresholds was assessed separately by delivery mode using sensitivity, specificity, positive predictive value, negative predictive value, and receiver operating characteristic analysis.

**RESULTS:**

Among 769 deliveries, postpartum anemia occurred in 22.0% (169/769), and severe postpartum anemia, defined as Hb<8 g/dL, occurred in 0.5% (4/769). In adjusted models, compared with QBL<500 mL, higher QBL categories were associated with increased odds of postpartum anemia: 500 to 999 mL, adjusted odds ratio (aOR) 5.13 (95% confidence interval [CI], 2.59–10.13; *P*<.001), 1000 to 1499 mL, aOR 6.10 (95% CI, 1.94–19.23; *P*=.002), and ≥1500 mL, aOR 14.42 (95% CI, 2.26–91.95; *P*=.005). Each 500-mL increase in QBL was associated with greater Hb decline (adjusted mean difference, 0.53 g/dL; 95% CI, 0.39–0.68; *P*<.001). Discrimination was modest in both delivery modes. The area under the receiver operating characteristic curve was 0.646 (95% CI, 0.571–0.721) for vaginal deliveries and 0.593 (95% CI, 0.523–0.663) for cesarean deliveries, with no significant difference between delivery modes (*P*=.313). At the ≥500-mL threshold, sensitivity was 22.4% (17/76) and specificity was 96.3% (394/409) for vaginal deliveries, compared with sensitivity of 90.3% (84/93) and specificity of 16.8% (32/191) for cesarean deliveries.

**CONCLUSION:**

Among patients without baseline anemia, higher QBL was associated with postpartum anemia and greater Hb decline; however, QBL alone had limited screening performance. Overall, the ≥500-mL threshold failed to identify 40.2% (68/169) of postpartum anemia cases, and its sensitivity and specificity varied markedly by delivery mode. Postpartum complete blood count testing decisions should be individualized and incorporate QBL alongside baseline Hb, maternal symptoms, delivery characteristics, and clinical judgment.

**AJOG Global Reports at a Glance**

**Why was this study conducted?**

To evaluate quantitative blood loss associations with postpartum anemia and hemoglobin decline and assess thresholds by delivery mode.

**Key findings**

Higher quantitative blood loss was associated with postpartum anemia and greater hemoglobin decline, but discrimination was modest. The ≥500-mL threshold missed 40.2% of postpartum anemia cases and differed by delivery mode, with low sensitivity after vaginal delivery and low specificity after cesarean delivery.

**What does this add to what is known?**

A single quantitative blood loss threshold does not perform consistently across delivery modes. Quantitative blood loss alone may be insufficient for screening and should be interpreted with baseline hemoglobin, maternal symptoms, delivery characteristics, and clinical judgment when determining the need for postpartum complete blood count testing.

## Introduction

Postpartum hemorrhage and postpartum anemia remain major contributors to maternal morbidity worldwide. Accurate assessment of blood loss at delivery is essential for timely recognition and management of hemorrhage and related complications. Visual estimation of blood loss has repeatedly shown poor accuracy, with a tendency toward misestimation across both vaginal and cesarean deliveries, prompting the adoption of objective measurement strategies.^1^

Accordingly, professional organizations recommend routine use of quantitative blood loss (QBL) during delivery to improve recognition of postpartum hemorrhage.^2^ Implementation studies suggest that QBL systems can standardize blood loss assessment and are feasible in routine obstetric practice.^3^ Despite these advances, uncertainty remains regarding how well QBL reflects downstream physiological outcomes such as hemoglobin (Hb) decline, postpartum anemia, and the need for blood transfusion.^4^ Most current literature evaluates QBL as an outcome or focuses on its role in hemorrhage recognition, rather than as a predictor of clinical hematologic outcomes.

In practice, postpartum Hb testing is commonly obtained to screen for anemia and to guide postpartum management.^5^ However, routine testing may not be necessary for all patients. Prior work has explored QBL-based thresholds to guide selective laboratory evaluation and reduce unnecessary phlebotomy in low-risk patients.^6^^,^^7^ In addition, institutional initiatives have evaluated omission of routine postpartum complete blood count (CBC), with one 2014 study reporting annual cost savings of approximately $58,000.^8^^,^^9^ Whether QBL is reliable for this purpose, particularly across different delivery modes and baseline Hb levels, remains unclear.

The aim of this study was to evaluate the association between QBL and postpartum hematologic outcomes, including anemia and Hb change, and assess the performance of commonly used QBL thresholds in a real-world clinical setting.

## Materials and methods

### Study design and population

This was a retrospective cohort study of patients who delivered at an urban safety-net hospital in the United States from January 1 to December 31, 2023. Eligible patients had singleton gestations, delivery at greater than 20 weeks of gestation, documented QBL, and both antepartum/baseline and postpartum Hb values available. Patients were excluded if they received a blood transfusion, had baseline anemia, or had conditions expected to substantially alter bleeding risk or Hb levels independent of delivery blood loss, including hemoglobinopathies, placenta previa or placenta accreta spectrum, coagulopathy, sepsis, multiple gestation, or major fetal anomalies. All transfusions occurred before postpartum Hb measurement. Baseline anemia was excluded so that postpartum Hb<10 g/dL would reflect anemia developing after delivery rather than preexisting anemia, and patients transfused before postpartum Hb measurement were excluded because transfusion would alter the measured postpartum Hb outcome.

### Quantitative blood loss measurement

QBL was calculated and documented by nursing staff according to the institutional standardized protocol. For vaginal deliveries, QBL measurement began immediately after delivery of the infant, with preplacental and amniotic fluid accounted for separately before blood loss quantification. Blood collected in the calibrated under-buttocks drape was combined with gravimetric assessment of blood-soaked sponges and laps when these materials were used, using standardized dry weights.

For cesarean deliveries, suction canister volume was adjusted for amniotic and irrigation fluid, and blood on surgical sponges and laps was incorporated using gravimetric assessment with standardized dry weights. QBL measurement ended at completion of the delivery or operative procedure. Nursing staff received standardized QBL training as part of labor and delivery orientation. Individual adherence to each component of the protocol was not independently audited for this retrospective study.

### Data collection

Clinical and demographic data were obtained from the electronic health record. Collected variables included maternal age, body mass index (BMI), gestational age at delivery, parity, delivery mode, labor induction or augmentation, hypertensive disorders of pregnancy, gestational diabetes mellitus, baseline Hb (from admission), and postpartum Hb values. Patient-level data on peripartum intravenous fluid administration were not available and therefore could not be included in the analysis.

### Outcomes

The primary outcome was postpartum anemia, defined as Hb<10 g/dL. Severe postpartum anemia, defined as Hb<8 g/dL, was evaluated descriptively. The Hb<10 g/dL cutoff was selected based on prior postpartum anemia literature.^4^ The Hb<8 g/dL cutoff was selected as a threshold for severe postpartum anemia based on prior published definitions.^10^ These postpartum outcome definitions were distinct from baseline anemia, which was assessed as a predelivery maternal characteristic and defined as Hb<11 g/dL. Anemia outcomes were determined using the routine postpartum day 1 CBC, typically scheduled for 5:00 am. Based on delivery times, the expected interval between delivery and CBC collection was approximately 5 to 29 hours; exact patient-level collection times were not available. Secondary outcomes included Hb decline from baseline and the diagnostic performance of selected QBL thresholds.

### Statistical analysis

Continuous variables were summarized as mean±standard deviation or median with interquartile range (IQR), as appropriate; categorical variables were reported as frequencies and percentages. Patients who received a blood transfusion before postpartum Hb measurement were excluded because transfusion could alter the measured postpartum Hb value. Hb change was calculated as baseline Hb minus postpartum Hb, so that positive values indicate a decline.

QBL was evaluated using four clinically relevant nominal categories: <500 mL, 500 to 999 mL, 1000 to 1499 mL, and ≥1500 mL, with <500 mL as the reference group. These thresholds were selected to reflect commonly used clinical blood loss categories, including traditional and contemporary thresholds for postpartum hemorrhage. Because proportional or linear effects across QBL strata could not be assumed, categories were modeled as nominal variables in the primary analyses. A secondary analysis modeled QBL as a continuous variable per 500 mL increment.

Multivariable logistic regression was used to estimate adjusted odds ratios (aORs) with 95% confidence intervals (CIs) for postpartum anemia (Hb<10 g/dL). Multivariable linear regression was used to estimate adjusted mean differences (*β* coefficients) in Hb decline. All models were adjusted a priori for baseline Hb, maternal age, BMI, parity, cesarean delivery, induction of labor, augmentation of labor, hypertensive disorder of pregnancy, and gestational diabetes mellitus. Given the small number of severe anemia events (*n*=4), adjusted regression for Hb<8 g/dL was not performed, and results for this outcome are presented descriptively. Complete-case analysis was used for adjusted models (*N*=707).

Diagnostic performance of QBL thresholds of ≥500 mL, ≥1000 mL, and ≥1500 mL was assessed separately for vaginal and cesarean deliveries using sensitivity, specificity, positive predictive value, and negative predictive value, each with 95% CIs calculated using the Wilson method. Receiver operating characteristic (ROC) analyses for postpartum anemia (Hb<10 g/dL) were performed separately by delivery mode, and the resulting areas under the curve were compared. Given the small number of severe postpartum anemia events, the ROC analysis for Hb<8 g/dL was performed in the pooled cohort and considered exploratory. The Youden index was used to identify the QBL value that maximized the combined sensitivity and specificity within each delivery mode.

QBL distributions were also summarized and graphically evaluated separately for vaginal and cesarean deliveries. In a secondary adjusted logistic regression model, an interaction term between continuous QBL, expressed per 500-mL increment, and delivery mode was evaluated. Potential heaping of QBL values was assessed descriptively by examining the frequency of values recorded at 50-mL and 100-mL increments.

All statistical tests were two-sided; a *P* value <0.05 was considered statistically significant. Statistical analyses were performed using PSPP (version 1.6.2; GNU Project). This manuscript was reported according to the Strengthening the Reporting of Observational Studies in Epidemiology (STROBE) guidelines for cohort studies. Institutional Review Board approval was obtained with a waiver of informed consent.

## Results

### Cohort characteristics

After sequential exclusion of patients with multiple gestation, baseline anemia, blood transfusion before postpartum Hb measurement, and missing QBL or postpartum CBC data, 769 patients were included in the final analytic cohort (Table 1). Of these, 62 were excluded from adjusted regression models because of missing covariate data, primarily BMI, yielding a complete-case analytic sample of 707 patients. Baseline cohort characteristics are shown in Table 2. Mean maternal age was 29.2±6.4 years, mean BMI was 32.8±6.3 kg/m², and mean baseline Hb was 12.1±0.8 g/dL. Median parity was 1 (IQR 0–2). Mean QBL was 416.5±352.5 mL. Postpartum anemia (Hb<10 g/dL) occurred in 169 patients (22.0%), and severe postpartum anemia (Hb<8 g/dL) occurred in 4 patients (0.5%).

**Table 1:** Derivation of the analytic cohort

Exclusion criterion	Excluded (*n*)	Remaining (*n*)
Total deliveries identified in 2023	—	1403
Multiple gestation	10	1393
Baseline anemia (hemoglobin<11 g/dL)	354	1039
Blood transfusion	7	1032
Missing quantitative blood loss or postpartum complete blood count	263	769
**Final analytic cohort**	**—**	**769**

Missing quantitative blood loss or postpartum complete blood count includes 258 records identified as incomplete in the source data system and 5 records with missing values identified within the study database. Arithmetic: 1403−10−354−7−263=769.

**Table 2:** Maternal, delivery, and hemoglobin characteristics of the analytic cohort (*N*=769)

Characteristic	Overall cohort (*N*=769)
Maternal age, y	29.2±6.4
Body mass index, kg/m²	32.8±6.3
***Race and ethnicity***	
Hispanic	538 (70.0%)
Black	222 (28.9%)
White	8 (1.0%)
Asian Indian	1 (0.1%)
Gestational age at delivery, wk	38.6±2.8
Parity	1 (0–2)
Baseline hemoglobin, g/dL	12.1±0.8
Postpartum hemoglobin, g/dL	10.9±1.2
Hemoglobin decline, g/dL	1.3±1.0
Quantitative blood loss, mL	416.5±352.5
Cesarean delivery	284 (36.9%)
Vaginal delivery	485 (63.1%)
Operative vaginal deliveryᵃ	43 (5.6%)
Induction of labor	104 (13.5%)
Augmentation of labor	116 (15.1%)
Gestational diabetes mellitus	99 (12.9%)
Hypertensive disorder of pregnancy	79 (10.3%)
Postpartum anemia, hemoglobin<10 g/dL	169 (22.0%)
Postpartum anemia, hemoglobin<8 g/dL	4 (0.5%)

Values are presented as mean±standard deviation, median (interquartile range), or *n* (%).

^a^Operative vaginal delivery includes 42 successful vacuum-assisted and 1 forceps-assisted deliveries and is included within the vaginal delivery category.

### Postpartum anemia by QBL category

Postpartum anemia was more frequent in higher QBL categories: 13.8% for QBL<500 mL, 36.2% for 500 to 999 mL, 36.0% for 1000 to 1499 mL, and 50.0% for ≥1500 mL (Table 3). In multivariable logistic regression with nominal QBL categories, compared with QBL<500 mL, the adjusted odds of postpartum anemia were significantly higher for QBL 500 to 999 mL (aOR 5.13, 95% CI 2.59–10.13), 1000 to 1499 mL (aOR 6.10, 95% CI 1.94–19.23), and ≥1500 mL (aOR 14.42, 95% CI 2.26–91.95; all *P*≤.005). In the secondary continuous model, each 500 mL increment in QBL was associated with a 3.09-fold increase in adjusted odds of postpartum anemia (95% CI 2.03–4.70; *P*<.001) (Table 4). In a secondary adjusted model, the association between continuous QBL and postpartum anemia did not differ significantly by delivery mode (*P* for interaction=.106).

**Table 3:** Postpartum outcomes by QBL category

QBL category, mL	*N*	Postpartum Hb<10 g/dL, *n*/*N* (%)	Postpartum Hb<8 g/dL, *n*/*N* (%)	Hb decline, g/dL, mean±SD
<500	494	68/494 (13.8%)	1/494 (0.2%)	1.07±0.94
500–999	240	87/240 (36.2%)	2/240 (0.8%)	1.66±1.07
1000–1499	25	9/25 (36.0%)	0/25 (0.0%)	1.82±1.01
≥1500	10	5/10 (50.0%)	1/10 (10.0%)	2.22±1.36

Hemoglobin decline=baseline hemoglobin minus postpartum hemoglobin; positive values indicate a decline.

*Hb*, hemoglobin; *QBL*, quantitative blood loss; *SD*, standard deviation.

**Table 4:** Adjusted associations of QBL with postpartum anemia and Hb decline

QBL exposure	Postpartum Hb<10 g/dL, aOR (95% CI)	*P* value	Adjusted *β* for Hb decline, g/dL (95% CI)	*P* value
Per 500 mL increase	3.09 (2.03–4.70)	<.001	0.53 (0.39–0.68)	<.001
500–999 vs <500 mL	5.13 (2.59–10.13)	<.001	0.64 (0.40–0.88)	<.001
1000–1499 vs <500 mL	6.10 (1.94–19.23)	.002	0.83 (0.39–1.27)	<.001
≥1500 vs <500 mL	14.42 (2.26–91.95)	.005	1.07 (0.42–1.72)	.001

*aOR*, adjusted odds ratio; *BMI*, body mass index; *CI*, confidence interval; *Hb*, hemoglobin; *QBL*, quantitative blood loss.

Complete-case *N*=707. All models adjusted for baseline hemoglobin, maternal age, BMI, parity, cesarean delivery, induction of labor, augmentation of labor, hypertensive disorder of pregnancy, and gestational diabetes mellitus. QBL categories were treated as nominal variables with <500 mL as reference. Severe anemia, defined as hemoglobin<8 g/dL, was not modeled in adjusted regression because of low number of events.

### Hb decline

Higher QBL was associated with greater Hb decline across all categories. In the nominal model, adjusted Hb decline was 0.64 g/dL greater for QBL 500 to 999 mL (95% CI, 0.40–0.88), 0.83 g/dL greater for 1000–1499 mL (95% CI, 0.39–1.27), and 1.07 g/dL greater for ≥1500 mL (95% CI, 0.42–1.72), each compared with QBL<500 mL (all *P*≤.001). In the continuous model, each 500 mL increase in QBL was associated with an adjusted 0.53 g/dL greater Hb decline (95% CI, 0.39–0.68; *P*<.001) (Table 4).

### QBL distribution and diagnostic performance

Mode-specific ROC analysis demonstrated modest discrimination for postpartum anemia (Hb<10 g/dL), with an area under the receiver operating characteristic curve (AUC) of 0.646 (95% CI, 0.571–0.721) for vaginal deliveries and 0.593 (95% CI, 0.523–0.663) for cesarean deliveries (Figure 1). The difference between the AUCs was not statistically significant (*P*=.313).

**Figure 1:**
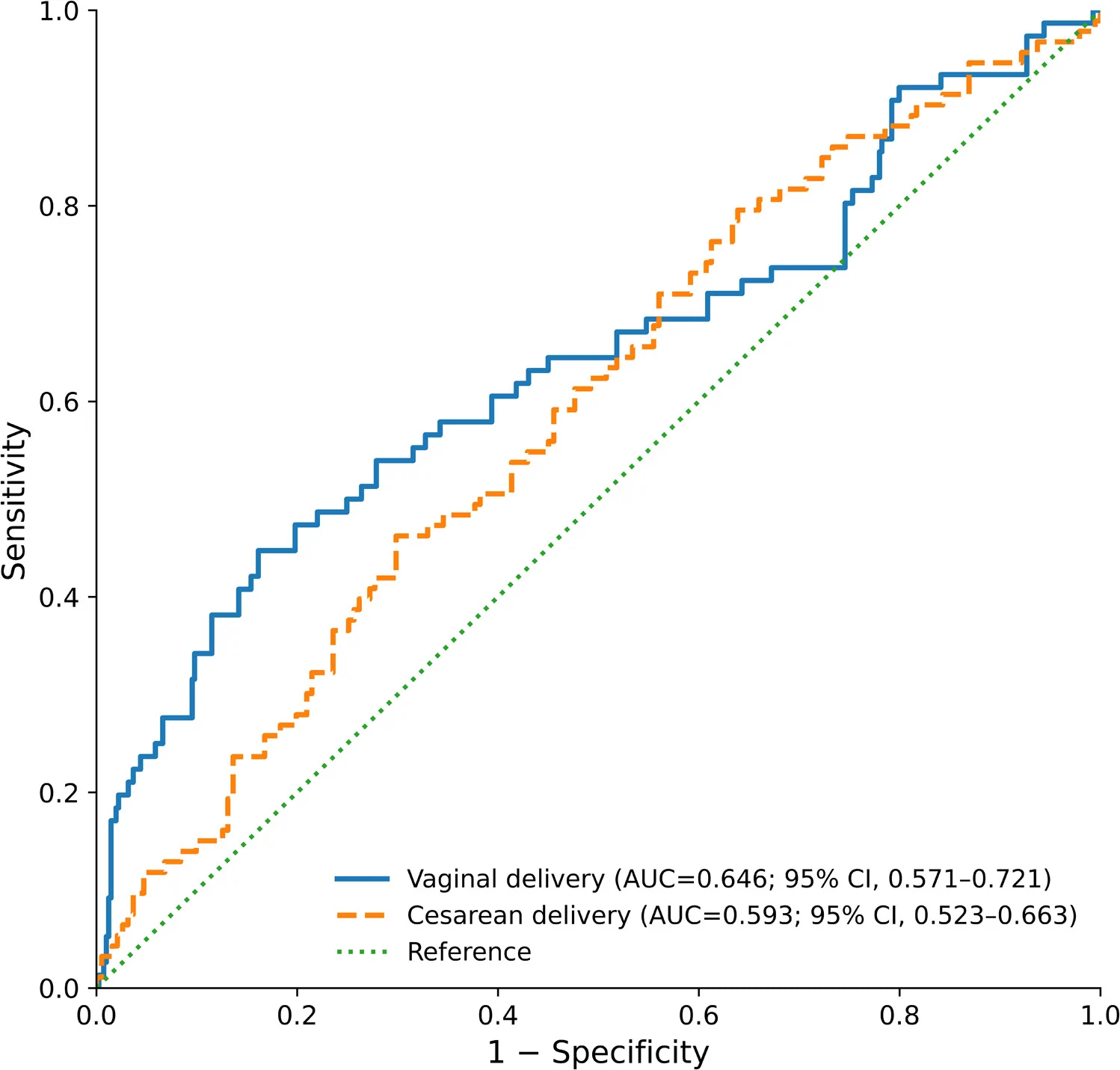
Receiver operating characteristic curves of quantitative blood loss for postpartum anemia by delivery mode Receiver operating characteristic curves for prediction of postpartum anemia, defined as hemoglobin<10 g/dL, using quantitative blood loss among vaginal and cesarean deliveries. The solid line represents vaginal delivery, the dashed line represents cesarean delivery, and the dotted diagonal line represents no discrimination. *AUC*, area under the receiver operating characteristic curve; *CI*, confidence interval.

Reyes Lizaola. Screening performance of quantitative blood loss for postpartum anemia among patients without baseline anemia. AJOG Glob Rep 2026.

QBL distributions differed substantially by delivery mode, with a median QBL of 150 mL (IQR, 100–289 mL; range, 0–1713 mL) after vaginal delivery and 745 mL (IQR, 571–894.5 mL; range, 24–1975 mL) after cesarean delivery (Figure 2). Descriptive assessment showed evidence of heaping primarily among vaginal deliveries: 24.7% of QBL values were recorded at 50-mL increments and 13.0% at 100-mL increments, compared with 3.2% and 1.4%, respectively, after cesarean delivery.

**Figure 2:**
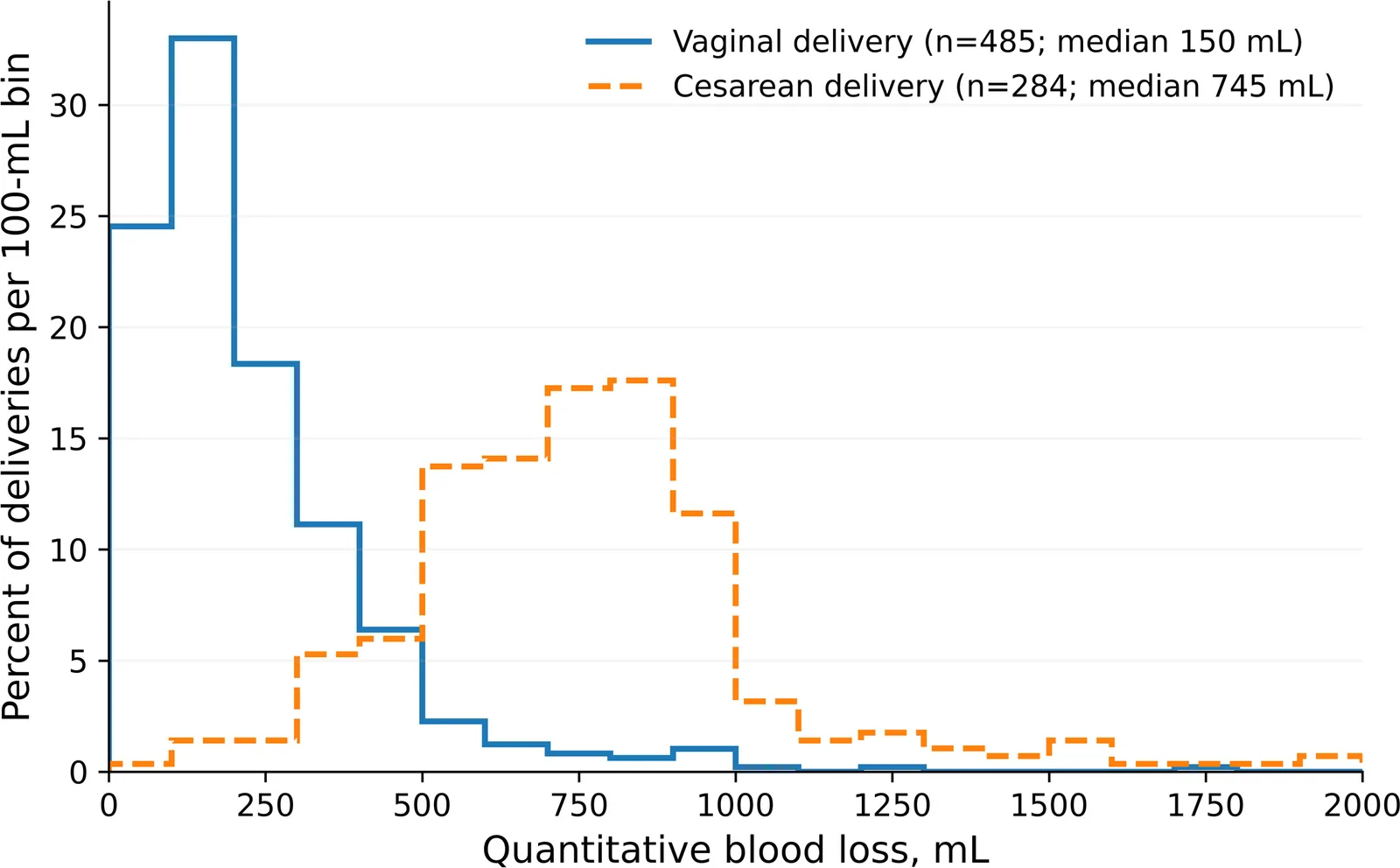
Distribution of quantitative blood loss by delivery mode Distribution of quantitative blood loss after vaginal and cesarean delivery, shown as the percentage of deliveries within 100-mL intervals. Percentages were calculated separately for each delivery mode. The solid line represents vaginal delivery, and the dashed line represents cesarean delivery.

Reyes Lizaola. Screening performance of quantitative blood loss for postpartum anemia among patients without baseline anemia. AJOG Glob Rep 2026.

The performance of fixed QBL thresholds also differed by delivery mode (Table 5). At the ≥500-mL threshold, sensitivity and specificity were 22.4% and 96.3%, respectively, for vaginal deliveries and 90.3% and 16.8%, respectively, for cesarean deliveries. When delivery modes were pooled, the ≥500-mL threshold failed to identify 68 of 169 patients with postpartum anemia (40.2%). Higher thresholds had greater specificity but low sensitivity in both delivery modes.

**Table 5:** Diagnostic performance of QBL thresholds for postpartum anemia (Hb<10 g/dL), by delivery mode

Delivery mode/QBL threshold	Cases detected	Sensitivity (95% CI)	Specificity (95% CI)	PPV (95% CI)	NPV (95% CI)
Vaginal ≥500 mL	17/76	22.4% (14.5–32.9)	96.3% (94.0–97.8)	53.1% (36.4–69.1)	87.0% (83.6–89.8)
Vaginal ≥1000 mL	1/76	1.3% (0.2–7.1)	99.5% (98.2–99.9)	33.3% (6.1–79.2)	84.4% (80.9–87.4)
Vaginal ≥1500 mL	0/76	0.0% (0.0–4.8)	99.8% (98.6–100.0)	0.0% (0.0–79.3)	84.3% (80.8–87.3)
Cesarean ≥500 mL	84/93	90.3% (82.6–94.8)	16.8% (12.1–22.7)	34.6% (28.9–40.7)	78.0% (63.3–88.0)
Cesarean ≥1000 mL	13/93	14.0% (8.4–22.5)	90.1% (85.0–93.5)	40.6% (25.5–57.7)	68.3% (62.3–73.7)
Cesarean ≥1500 mL	5/93	5.4% (2.3–12.0)	97.9% (94.7–99.2)	55.6% (26.7–81.1)	68.0% (62.3–73.2)

Cases detected are presented as *n*/*N*, where *N* is the total number of postpartum anemia cases within each delivery mode (76 vaginal deliveries and 93 cesarean deliveries).

*CI*, confidence interval; *Hb*, hemoglobin; *NPV*, negative predictive value; *PPV*, positive predictive value; *QBL*, quantitative blood loss.

Exploratory Youden-index thresholds were 314 mL for vaginal delivery, with sensitivity of 44.7% and specificity of 83.9%, and 850 mL for cesarean delivery, with sensitivity of 46.2% and specificity of 70.2%. These data-derived thresholds were exploratory and were not considered validated clinical cutoffs. For severe postpartum anemia (Hb<8 g/dL), the pooled AUC was 0.843; however, because this outcome occurred in only 4 patients, the analysis was exploratory and should be interpreted with caution.

## Discussion

### Principal findings

In this retrospective cohort study, higher QBL at delivery was associated with increased odds of postpartum anemia and greater Hb decline. These associations persisted after adjustment for baseline Hb and obstetric covariates, supporting QBL as a marker of postpartum hematologic outcomes. At the same time, these findings highlight an important limitation: QBL has limited performance as a stand-alone screening measure to guide postpartum testing or management decisions.

### Results

A key finding of this study is that although higher QBL was associated with adverse hematologic outcomes at a population level, commonly used QBL thresholds had limited sensitivity for identifying patients who developed postpartum anemia. This observation is consistent with prior studies demonstrating imperfect correlation between measured blood loss and postpartum Hb change or anemia severity.^4^^,^^11^ Blood loss at delivery represents only one component of a complex physiological process that reflects intravascular volume shifts, baseline iron status, and the timing of laboratory assessment.^6^^,^^7^^,^^12^^,^^13^ As a result, patients with similar measured blood loss may experience substantially different postdelivery Hb values.

The graded association between higher QBL categories and postpartum anemia supports the validity of QBL as a clinically meaningful exposure. However, CIs widened at higher QBL categories, likely reflecting smaller sample sizes and greater clinical heterogeneity among patients with large-volume blood loss. These findings suggest that while increasing QBL generally tracks with worsening outcomes, the precision of risk estimates diminishes at higher blood loss values.

The performance characteristics of QBL thresholds differed by delivery mode and highlight an important trade-off between sensitivity and specificity. Although QBL may facilitate earlier recognition of hemorrhage and shorter time to transfusion,^14^ the ≥500-mL threshold had low sensitivity and high specificity after vaginal delivery, whereas it had high sensitivity and low specificity after cesarean delivery. At higher thresholds, specificity increased, but sensitivity was low in both delivery modes. Similar concerns have been raised in studies evaluating QBL-based screening strategies, suggesting that reliance on QBL alone may miss cases of postpartum anemia.^6^^,^^7^ In practice, the absence of a high QBL value should not be interpreted as reassurance that postpartum anemia will not occur.

The vaginal-delivery analysis further illustrates the limitations of using QBL alone to guide postpartum Hb testing. Among vaginal deliveries with QBL<500 mL, postpartum anemia occurred in 59 of 453 patients (13.0%). These findings suggest that QBL below 500 mL does not exclude postpartum anemia.

### Clinical implications

Overall, these findings have practical implications for postpartum Hb screening. Prior studies have evaluated selective postpartum laboratory testing strategies using criteria such as preoperative anemia, delivery characteristics, and measured blood loss, with mixed results.^5^, ^6^, ^7^, ^8^ These findings suggest that reliance on QBL alone to guide omission of postpartum Hb testing may miss patients with postpartum anemia. ROC findings further support this conclusion, as QBL alone demonstrated modest discrimination for postpartum anemia, indicating limited ability to classify individual patients accurately.

### Research implications

Future studies are needed to assess whether combining QBL with baseline Hb, symptoms, delivery characteristics, and other clinical factors improves the prediction of postpartum anemia compared with QBL alone.

### Strengths and limitations

Strengths of this study include the use of routinely collected QBL data from a real-world clinical setting, availability of paired baseline and postpartum Hb values, adjustment for key obstetric covariates, assessment of clinically relevant QBL thresholds using diagnostic performance measures, and delivery-mode-specific analyses including formal interaction testing.

Limitations include the retrospective design, single-center setting, potential selection bias due to missing QBL or Hb values, and variability in timing of postpartum Hb measurement. Because patients with baseline anemia, those who received transfusion before postpartum Hb measurement, and those with conditions expected to substantially alter bleeding risk or Hb levels were excluded, findings may not apply to patients at highest risk for postpartum anemia or severe hemorrhage-related morbidity.

The study focused on hematologic outcomes and did not assess maternal symptoms, postpartum iron treatment, transfusion after postpartum Hb measurement, or readmission, and the findings therefore do not address subsequent management or clinical outcomes related to postpartum anemia. QBL was obtained using a standardized institutional protocol; however, adherence to each component of the protocol was not independently audited. In addition, QBL values were more frequently recorded at common increments after vaginal delivery.

Although delivery-mode-specific analyses and formal interaction testing were performed, the exploratory Youden-index thresholds were derived from this single-center cohort and were not externally validated. Additionally, exact patient-level timing of postpartum Hb measurement and peripartum intravenous fluid administration was not available. Because Hb values can change over the early postpartum period as plasma volume shifts, measured Hb decline may not directly correspond to blood loss, and this should be considered when interpreting results.^15^, ^16^

## Conclusions

Despite these limitations, among patients without baseline anemia, these findings provide clinically relevant evidence that QBL is best used as one component of a broader decision framework rather than as the sole determinant of postpartum management. Higher QBL at delivery was associated with postpartum anemia; however, QBL demonstrated only modest discrimination, and the performance of fixed thresholds differed by delivery mode.

These findings suggest that QBL alone is insufficient to determine which patients can forgo postpartum CBC testing. QBL may contribute to risk assessment, but postpartum testing decisions should be individualized and incorporate baseline Hb, maternal symptoms, delivery characteristics, and clinical judgment.

## CRediT authorship contribution statement

**Sebastian Reyes Lizaola:** Writing – review & editing, Writing – original draft, Validation, Project administration, Methodology, Investigation, Data curation, Conceptualization. **Ivy Huynh:** Writing – original draft, Data curation. **Lucia Di Francesco:** Writing – review & editing, Visualization, Validation, Software, Methodology, Formal analysis, Data curation, Conceptualization. **Swati Kumari:** Writing – original draft, Investigation, Conceptualization. **Tajudeen Dabiri:** Writing – review & editing, Supervision, Methodology, Conceptualization.
